# Pluriannual experience in stapled haemorrhoidopexy in the elderly

**DOI:** 10.1186/1471-2318-11-S1-A60

**Published:** 2011-08-24

**Authors:** S Spirch, F Tona, C Sperti, M Gruppo, F Mazzalai, R Lorenzetti, M Di Giunta, C Sirianni, O Terranova

**Affiliations:** 1 Dipartimento di Scienze Chirurgiche e Gastroenterologiche, Clinica Chirurgica Geriatria, Università di Padova, Italy; 2 Dipartimento di Scienze Mediche e Chirurgiche, Clinica Chirurgica IV, Università di Padova, Italy; 3 Senior House Officer, Medicine and A&E Directorate, Leicester Royal Infirmary, UK

## Background

Compare two groups of patients, ≥70 years old and <70 years old, diagnosed with III-IV grade haemorrhoids that underwent to stapled haemorrhoidopexy [1].

## Materials and methods

Between May 2001 and August 2010, 320 patients underwent stapled haemorrhoidopexy (PPH Ethicon-EndoSurgery®). The database has been organised into two groups: the first composed of 30 patients (9.3%) aged≥70, while the second composed of 290 patients aged<70. The preferred type of anaesthesia was spinal with sedation (92.8%), combined with elastomeric pump of NSAIDs during the first 24 hours. The two groups were compared in order to verify their homogeneity: no significant differences were found either in the distribution of the grade of the disease, or in the spectrum of symptoms (P>>0.05). Because of the comorbidity in the elderly, the stratification of the ASA risk was different (P<0.0001).

## Results

The procedure was performed in day surgery, with an average length of stay of 1 day, in 75% of the patients of the first group and in the 92% of the second group; the analysis of the surgical performance of this technique, regarding the timing and the use of haemostatic stitches, showed no significant differences between the two groups (P>>0.05). Early haemorragic post-operative complications were 2.3%, of which 1 occurred in the first group and 6/7 required surgical review. There were 10 late haemorragic complications, all of them occurred in the second group; the management of the late haemorrhages required surgical review in 40% of patients and blood transfusion in 30% of cases. During the first post-operative week several cases of significant anal pain occurred, tenesmus, faecal urgency and two cases of haemorrhoidal thrombosis. During the follow-up, which lasted on average for 4.1 years, we observed 3 relapses (10%) among the first group and 21 relapses (7.2%) among the second.

## Conclusions

Haemorrhoidal disease, although tending to relapse among susceptible patients, can be effectively treated with stapled haemorrhoidopexy (PPH). Thanks to a several years’ follow-up, our experience shows an assessment of the long-term results of this technique, focusing particularly on the comparison between the results in the elderly and in younger patients. The Longo technique is usually well tolerated by all the patients, even though not totally pain-free in the early post-operative follow-up (first week). This procedure can be performed safely in the elderly as well as in the younger patients with equivalent results.
